# P041 Central retinal artery occlusion as the initial manifestation of giant cell arteritis in the absence of cranial symptoms

**DOI:** 10.1093/rap/rkag113.071

**Published:** 2026-11-03

**Authors:** Rehab Mohammed, Kirsty Levasseur

**Affiliations:** Rheumatology Department, The Dudley Group, Dudley, United Kingdom; Rheumatology Department, The Dudley Group, Dudley, United Kingdom

## Abstract

**Introduction:**

Central retinal artery occlusion (CRAO) is a rare ophthalmic emergency and a cause of irreversible visual loss. It may represent the initial manifestation of giant cell arteritis (GCA), even in the absence of classical cranial symptoms such as headache, scalp tenderness, or jaw claudication. Prompt recognition and early vascular imaging are essential to prevent further morbidity. We report a case of GCA presenting with CRAO without preceding cranial features, confirmed on temporal artery ultrasound.

**Case description:**

A 70-year-old gentleman was referred with a five-day history of inflammatory-type polyarthralgia affecting the left wrist and metacarpophalangeal joints, associated with swelling and reduced grip strength. He had no history of psoriasis, inflammatory bowel disease, or established inflammatory arthritis.

He concurrently reported acute right eye visual loss, diagnosed as central retinal artery occlusion resulting in permanent monocular blindness. He denied headache, scalp tenderness, jaw claudication, or other symptoms suggestive of GCA. Examination revealed synovitis of the left wrist and MCP joints. Blood tests showed elevated inflammatory markers (ESR 77 mm/hr, CRP 55 mg/L). Rheumatoid factor, anti-CCP antibodies, ANA, and uric acid were negative. Isolated anti–β2 glycoprotein I antibody positivity was identified.

Given ocular ischaemia with systemic inflammation, urgent temporal artery ultrasound demonstrated a halo sign in the left parietal branch, consistent with giant cell arteritis. High-dose prednisolone (60 mg daily) was commenced with tapering.

Joint symptoms improved following treatment, and imaging demonstrated features consistent with calcium pyrophosphate deposition disease contributing to intermittent inflammatory arthropathy.

During follow-up, the patient developed right hip pain. MRI confirmed subchondral fractures consistent with avascular necrosis of the femoral head, likely related to corticosteroid therapy. He remains under orthopaedic review and continues on a structured steroid taper with bone protection therapy.

**Discussion:**

This case highlights central retinal artery occlusion as the initial manifestation of GCA in the absence of cranial symptoms. CRAO is a sight-threatening emergency that should prompt urgent evaluation for GCA, even when classical features are absent.

In this case, the combination of ocular ischaemia and systemic inflammation led to early vascular imaging, and temporal artery ultrasound provided rapid diagnostic confirmation, enabling immediate treatment initiation. This supports the growing role of ultrasound as a first-line diagnostic tool in atypical GCA presentations.

The presence of inflammatory polyarthralgia initially broadened the differential diagnosis, with subsequent imaging demonstrating calcium pyrophosphate deposition disease. This acted as a diagnostic confounder but did not delay vascular assessment once ocular ischaemia was identified.

An isolated anti–β2 glycoprotein I antibody was detected. Its clinical significance in the context of arterial occlusion remains uncertain, and no formal antiphospholipid syndrome diagnosis was made.

Long-term corticosteroid therapy was complicated by avascular necrosis of the femoral head, highlighting the importance of vigilance for treatment-related morbidity and the need for bone protection strategies and orthopaedic involvement in follow-up care.

Overall, this case reinforces that CRAO may be the presenting feature of GCA without cranial symptoms, and that rapid vascular imaging is critical to prevent diagnostic delay and irreversible visual loss.

**Key learning points:**

1. Central retinal artery occlusion may be the initial manifestation of GCA without cranial symptoms.

2. Absence of classical cranial features does not exclude GCA in patients presenting with ocular ischaemia.

3. Temporal artery ultrasound enables rapid, non-invasive confirmation in atypical presentations of GCA.

4. Isolated anti–β2 glycoprotein I antibody positivity may be present in vascular presentations, but its clinical significance remains uncertain.

5. Corticosteroid therapy may be complicated by avascular necrosis, requiring ongoing monitoring and multidisciplinary management.

